# Path planning for the Platonic solids on prescribed grids by edge-rolling

**DOI:** 10.1371/journal.pone.0252613

**Published:** 2021-06-02

**Authors:** Ngoc Tam Lam, Ian Howard, Lei Cui

**Affiliations:** School of Civil and Mechanical Engineering, Curtin University, Bentley, WA, Australia; Consiglio Nazionale delle Ricerche, ITALY

## Abstract

The five Platonic solids—tetrahedron, cube, octahedron, dodecahedron, and icosahedron—have found many applications in mathematics, science, and art. Path planning for the Platonic solids had been suggested, but not validated, except for solving the rolling-cube puzzles for a cubic dice. We developed a path-planning algorithm based on the breadth-first-search algorithm that generates a shortest path for each Platonic solid to reach a desired pose, including position and orientation, from an initial one on prescribed grids by edge-rolling. While it is straightforward to generate triangular and square grids, various methods exist for regular-pentagon tiling. We chose the Penrose tiling because it has five-fold symmetry. We discovered that a tetrahedron could achieve only one orientation for a particular position.

## Introduction

The history of the Platonic solids—tetrahedron, cube, octahedron, dodecahedron, and icosahedron—can be traced back to over 2000 years ago. In ancient Greece, Pythagoras (c.570-c.495 BC) knew of the tetrahedron, cube, and dodecahedron [1]. Plato (c.427–347 B.C), to whom the names of these five regular polyhedra are attributed, assigned them to the four basic elements—fire, air, water, and earth—as well as the heavens [2]. In the 1600s, Kepler proposed a model of the solar system consisting of the Platonic solids set inside one another, distanced by the inscribed and circumscribed spheres of each solid [3]. The Platonic solids are all convex polyhedra bounded by a finite number of regular polygons. Being highly symmetric, the Platonic solids have found many applications in mathematics, science, and art. For example, in studying molecules, they were used to predict material structures of crystals [4] or to reconstruct colloidal crystals from symmetric hard particles [5]. In mathematics, each Platonic solid was used for a 3D billiard table to model how a cue ball moves to hit every face and return to its starting point [6].

Planning techniques are categorized into different aspects. The basic idea of discrete path planning in the most cases is that state-space models will be used to demonstrate the distinct situation in which the task of a planning algorithm solves the sequence actions transforming from a initial state to other states [7]. For example, Thomas [8] applied Delaunay triangulations to discretize the environment, and cubic spline representations are proposed to meet robot kinematic constraints. Considering the continuous curvature on smooth curves has been integrated within the probabilistic approaches in order to compute the piecewise smooth paths for a car-like vehicle as a four-dimensional system [9]. Whereas, dealing with nonholonomic constraints, a sampling-based road map technique was proposed in [10]. Based on decomposing space into cells [11], a potential field without local minima was assigned with polygonal partitions of planar environments to solve the Laplace’s equation problems in each cell exist.

The fundamental robotic path planning problem is to represent the environment as a graph involving the set of possible robot location and a set of edges that can generate the paths. The popular method for determining the least-cost paths is A* as Heuristic based search algorithm in [12–14]. The search algorithm must expand the fewest possible nodes in order to make searching for an admissible path. Then the evaluation of available nodes is needed to determine the next efficient nodes. The initial search approached by A* takes two steps to generate an optimal path in which receiving information from one of the initial cells in free space and replanning from scratch when the environment has changed to expand a new cell. However, the A* computation process needs high configuration processors to successfully reach various nodes. In the real world scenarios, the search operating sometimes may be performed with inaccurate planning graphs.

Literature on path planning for polyhedra by edge-rolling is scarce. An attempt was made to plan a path for an octahedron edge-rolling on a plane from an initial pose (position and orientation) to a desired pose, which, unfortunately, failed due to errors propagating from the algorithm [15]. In graspless manipulation, two movable parallel plates working as a robotic end-effector rolled a cubic dice by edges [16], but this work did not discuss how to generate the desired path. The rolling-cube puzzles, which focus on how to roll a cubic dice on a board consisting of labeled and white cells, were solved by detecting a Hamiltonian path in grid graphs as an NP-hard problem [17]. In this work, we propose a path-planning algorithm using tree exploration for each of the five Platonic solids starting from an initial pose to a desired pose by edge-rolling on different prescribed grids. We believe this is the first work that solves this problem.

This article is organized as follows. Firstly, the geometrical parameters for the Platonics solids and the different patterns of grids are briefly reviewed. Secondly, the path planning algorithm is described. Then, the simulations for the proposed algorithm is presented. Finally, this article is concluded.

## Background

This section provides an overview of the properties of the Platonics solids and different patterns of grids used to implement a path planning algorithm.

### Properties of polyhedron

**Fig 1:**
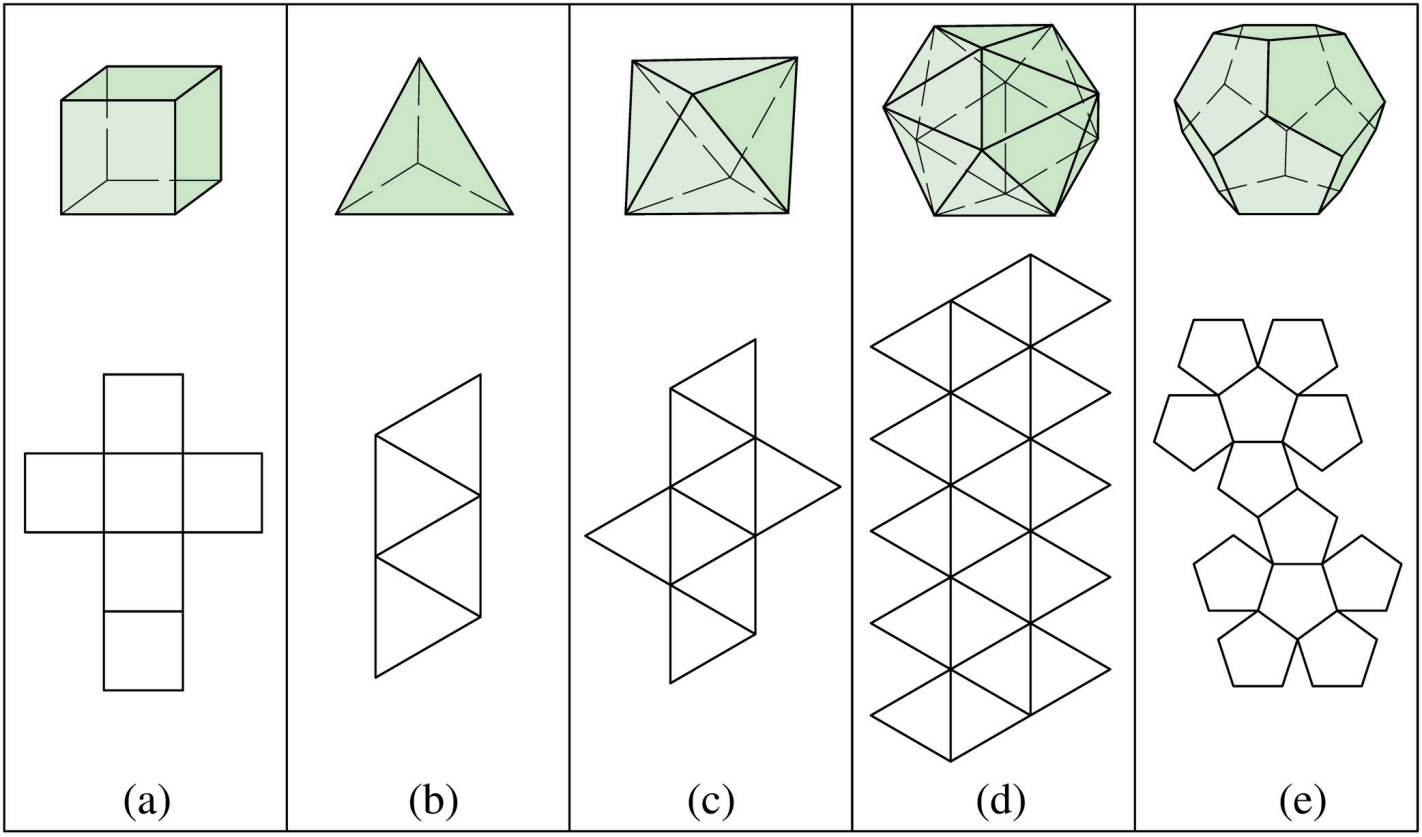
Five models of the Platonic solids and their unfolding geometry. (a) Cube. (b) Tetrahedron. (c) Octahedron. (d) Icosahedron. (e) Dodecahedron.

Each of the Platonic solids can be unfolded into non-overlapping edge-joining polygons (Fig 1). The cube is constructed by 6 squares; the tetrahedron consists of 4 equilateral triangles joined at their edges into a triangular pyramid; the octahedron has a double-pyramid structure with 8 equilateral triangles; the icosahedron has 20 equilateral triangles; and the dodecahedron is composed of 12 regular pentagons (Table 1). The total number of vertices (*V*), edges (*E*), and faces (*F*) of the Platonic solids satisfy Euler’s formula: *V* − *E* + *F* = 2 [18].

**Table 1:** Properties of polyhedron.

Platonic solids	Faces (*F*)	Edges (*E*)	Vertices (*V*)	Edges on each face (*e*_*f*_)	Faces meeting at each vertex (*f*_*v*_)
**Tetrahedron**	4	6	4	3	3
**Cube**	6	12	8	4	3
**Octahedron**	8	12	6	3	4
**Icosahedron**	20	30	12	3	5
**Dodecahedron**	12	30	20	5	3

### Discretized grids

**Fig 2:**
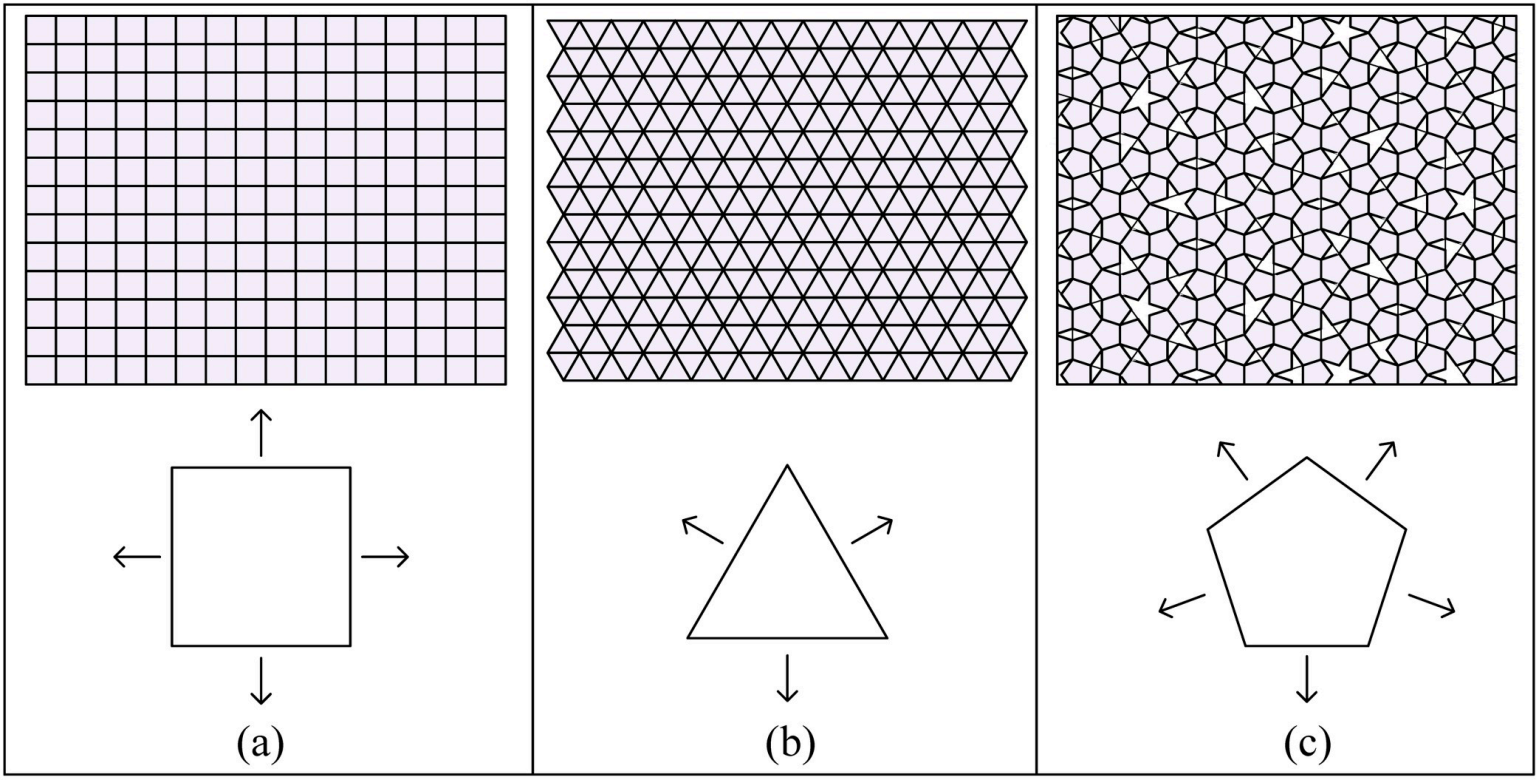
Direction of grids. Three different patterns of grid of the plane for the Platonic solids. (a) A square grid for the cube with 4 edge-rolling directions. (b) A triangular grid for the tetrahedron, octahedron, and icosahedron with 3 edge-rolling directions. (c) A pentagon grid using Penrose tiling for the dodecahedron with 5 edge-rolling directions.

A plane can be discretized into a square, triangular, or pentagon grid, depending on the face of a Platonic solid in contact with it (Fig 2). At any instant, a cube has 4 edges in contact with the plane, which indicates 4 possible directions of edge-rolling on the square grid; a tetrahedron, octahedron, or icosahedron has 3 edges in contact with the plane, which indicates 3 possible directions of edge-rolling on the triangular grid; a dodecahedron has 5 edges in contact with the plane, which indicates 5 possible directions of edge-rolling on the pentagon grid.

**Fig 3:**
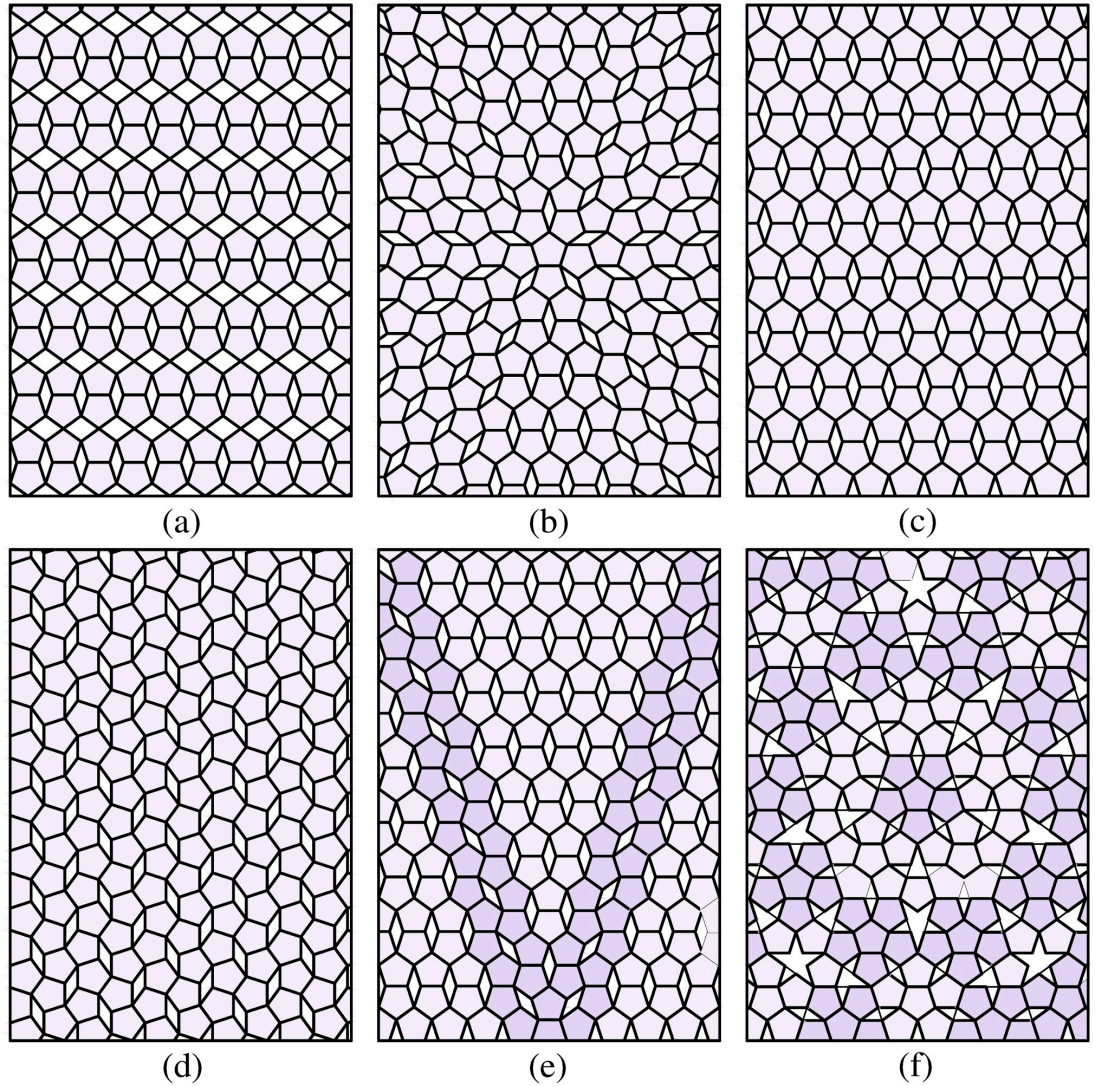
Patterns of the regular pentagon tiling. (a), (b) Two patterns of pentagon tiling from Durer including a five-fold nucleus that is expanded by multiple twins of five-fold symmetry (reconstructed from [6]). (c)-(e) Three patterns of pentagon tiling in art proposed by Caris (reconstructed from [7]). (f) Penrose tiling with five-fold symmetry generated by attaching multiple groups of a pentagon to the initial one (reconstructed from [8]).

**Fig 4:**
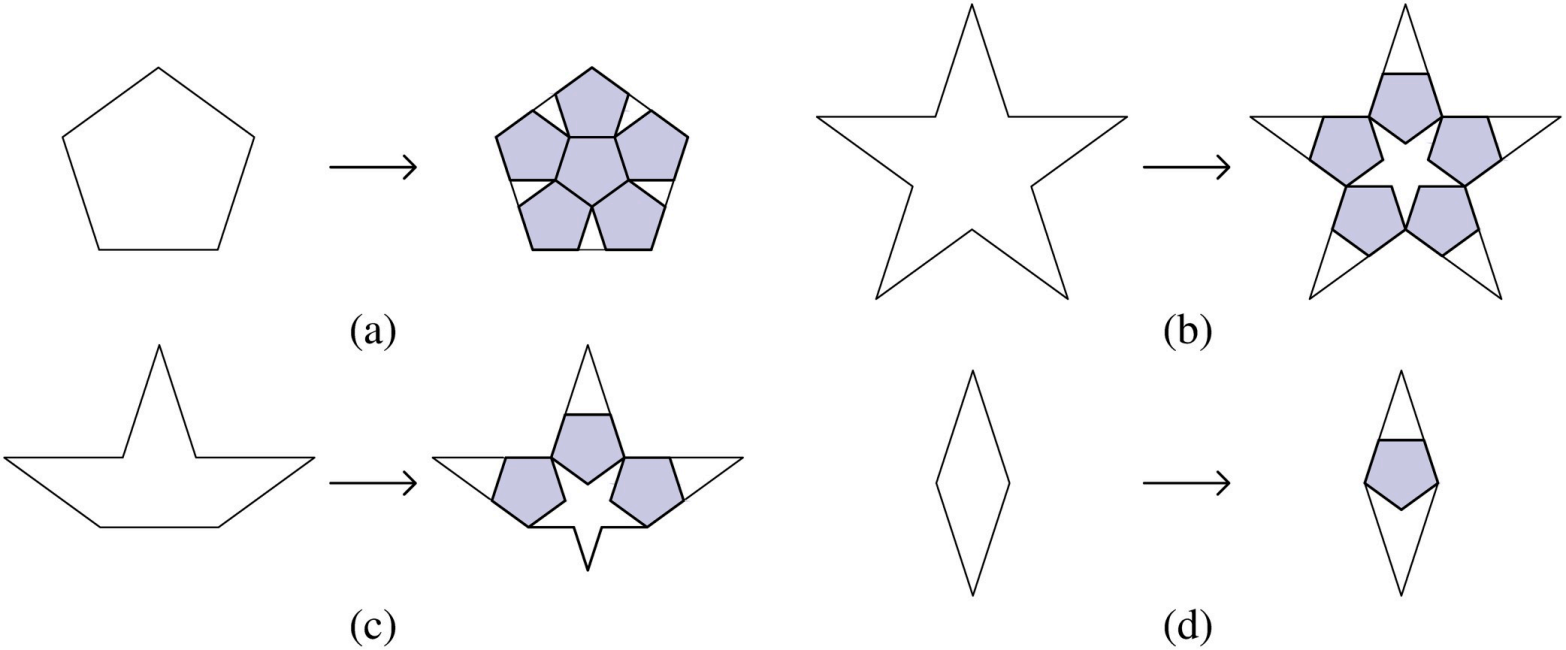
Substitution rules for Penrose tiling (reconstructed from [9]). (a) A pentagon is partially filled by 6 pentagons. (b) A pentacle is partially filled by 5 pentagons. (c) A half-pentacle is partially filled by 3 pentagons. (d) A rhombus is partially filled by 1 pentagon.

There are many options for discretizing a plane into a pentagon grid. Regular pentagons tiling a plane will leave symmetric gaps without overlap (Fig 3). A variety of patterns exist, such as those developed by Dürer (Fig 3(a) and 3(b)) [19], Caris (Fig 3(c)–3(e)) [20], and Penrose (Fig 3(f)) [21]. The Dürer and Caris tiling uses multiple twins of regular pentagons to tile a plane, in which rhombi remain between pentagons in various positions. The Penrose tiling attaches five regular pentagons onto the initial one along its edges to form a new larger pentagon, which generates gaps in the shapes of rhombi, pentacles, and half-pentacles. These gaps are partially filled by inserting pentagons following substitution rules (Fig 4) [22]. To facilitate path planning, we chose Penrose tiling because it has five-fold symmetry, which others lack.

### Rotation matrix

In this paper, the Rodrigues’ rotation matrix from the axis-angle representation [23] was used to calculate the orientation of a Platonic solid from a current position to next position after edge-rolling. A unit vector $\omega \in R^{3}$ and a rotation angle *β*, which are specific with respect to each Platonic solid, were used to generate a rotation matrix ***R*** ∈ *SO*(3) (Eq (1)). The coordinates of the Platonic solids before and after edge-rolling were represented by matrices ***M*** and ***M***′, where ***M***′ = ***MR***, respectively. In a three-dimensional Euclidean coordinate system, an axis-angle representation is given by a unit vector $\omega =\left(\omega_{x},\omega_{y},\omega_{z}\right)\in R^{3}$ and an angle *β*. The rotation angle *β* of each Platonic solid is the supplementary angle of the dihedral angle *α*, which is the angle between two intersecting faces, as in Table 2. The rotation matrix ***R*** can be obtained as:

$$\begin{array}{cc} R & =e^{\beta S_{\omega }}\\ & =I+sS_{\omega }+\left(1-c\right){S_{\omega }}^{2}\\ & =[\begin{array}{ccc} {\omega_{x}}^{2}+\left(1-{\omega_{x}}^{2}\right)c & \omega_{x}\omega_{y}\left(1-c\right)-\omega_{z}s & \omega_{x}\omega_{z}\left(1-c\right)+\omega_{y}s\\ \omega_{x}\omega_{y}\left(1-c\right)+\omega_{z}s & {\omega_{y}}^{2}+{(1-\omega_{y}}^{2})c & \omega_{y}\omega_{z}\left(1-c\right)-\omega_{x}s\\ \omega_{x}\omega_{z}\left(1-c\right)-\omega_{y}s & \omega_{y}\omega_{z}\left(1-c\right)+\omega_{x}s & {\omega_{z}}^{2}+\left(1-{\omega_{z}}^{2}\right)c \end{array}] \end{array}$$ (1)

where *s* = sin*β*, *c* = cos*β*, the 3 × 3 identity matrix ***I***, and the following skew-symmetric matrix ***S***_***ω***_ (2):

$$\begin{array}{c} \begin{array}{c} S_{\omega }=[\begin{array}{ccc} 0 & -\omega_{z} & \omega_{y}\\ \omega_{z} & 0 & -\omega_{x}\\ -\omega_{y} & \omega_{x} & 0 \end{array}] \end{array} \end{array}$$ (2)

**Table 2:** Geometrical parameters of the Platonic solids with inradius (*r*_*i*_), midradius (*r*_*m*_), circumradius (*R*), dihedral angles (*α*) and rolling angles (*β*). In this table, all the edges of the Platonic solids have the same unit length (*l* = 1).

Platonic solids	*r*_*i*_	*r*_*m*_	*R*	*α*	*β*
**Tetrahedron**	$\frac{\sqrt{6}}{12}$	$\frac{\sqrt{2}}{4}$	$\frac{\sqrt{6}}{4}$	$\arccos(\frac{1}{3})$	$2\arctan(\sqrt{2})$
**Cube**	$\frac{1}{2}$	$\frac{\sqrt{2}}{2}$	$\frac{\sqrt{3}}{2}$	$\frac{\pi }{2}$	$\frac{\pi }{2}$
**Octahedron**	$\frac{\sqrt{6}}{6}$	$\frac{1}{2}$	$\frac{\sqrt{2}}{2}$	$\arccos(-\frac{1}{3})$	$\arccos(\frac{1}{3})$
**Icosahedron**	$\frac{1}{12}\left(3\sqrt{3}+\sqrt{15}\right)$	$\frac{1}{4}\left(1+\sqrt{5}\right)$	$\frac{1}{4}\sqrt{10+2\sqrt{5}}$	$\arccos(-\frac{\sqrt{5}}{3})$	$\arccos(\frac{\sqrt{5}}{3})$
**Dodecahedron**	$\frac{1}{2}\sqrt{\frac{1}{10}\left(25+11\sqrt{5}\right)}$	$\frac{1}{4}\left(3+\sqrt{5}\right)$	$\frac{1}{4}\left(\sqrt{15}+\sqrt{3}\right)$	$\arccos(-\frac{\sqrt{5}}{5})$	$\arccos(\frac{\sqrt{5}}{5})$

## Path planning algorithm

The proposed algorithm generates paths for each Platonic solid edge-rolling from an initial pose to a desired pose on a plane. While at rest, a face of a Platonic solid is in contact with the plane, and the algorithm determines the edge, and subsequently the direction, of rolling.

### The tree exploration

**Fig 5:**
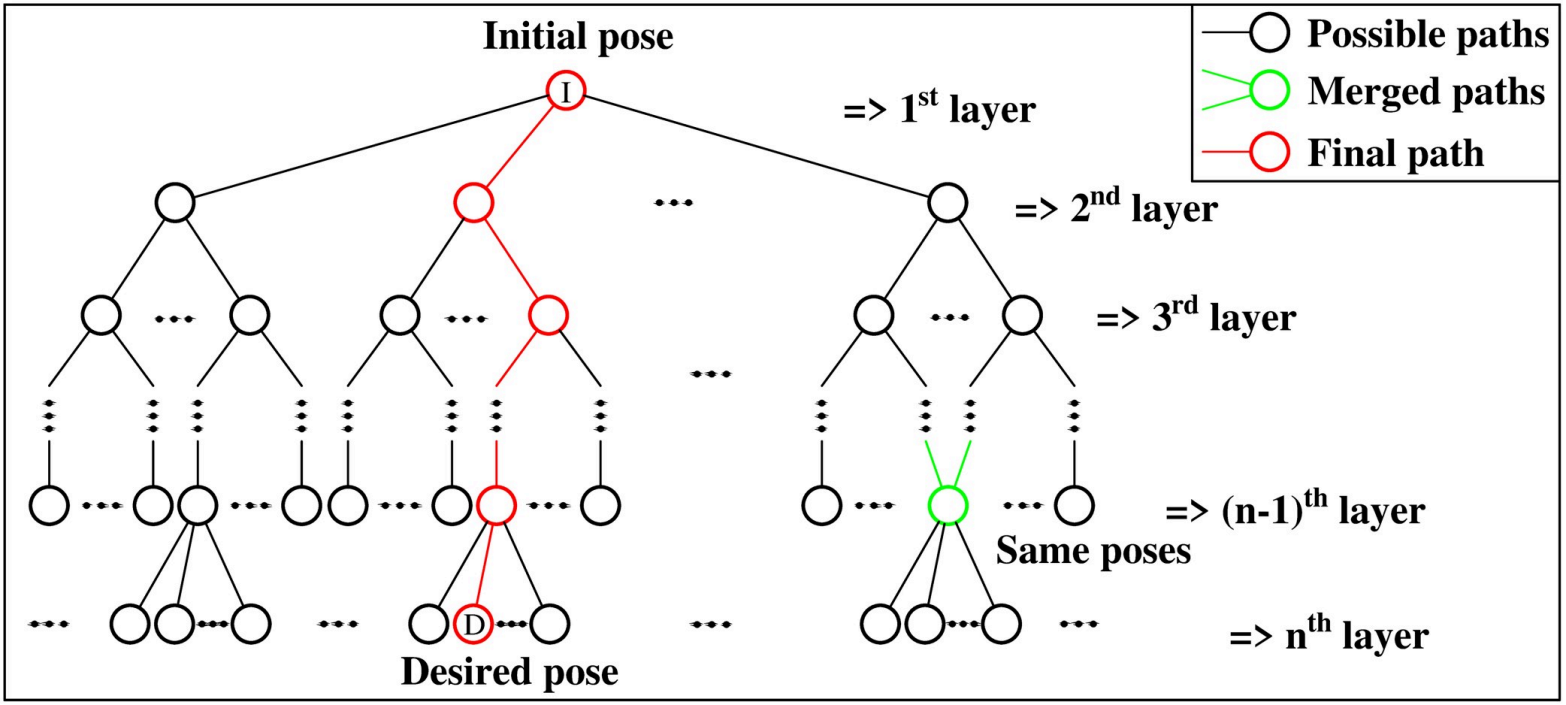
Tree exploration technique. Based on the BFS method, this algorithm starts from an initial pose represented by the Node I. The branches represent the rolling directions for each iteration. If some nodes are of the same pose, they are merged to reduce the search space (the green node). The algorithm stops when the desired pose, represented by the Node D, is reached and a shortest path is generated (colored in red).

The planning algorithm employs tree exploration (Fig 5), which is a variation of the breadth-first-search (BFS) algorithm [24]. Using queues, this algorithm is faster than the *A*^⋆^ algorithm, which uses the priority queue [25], for the unweighted graph. Another advantage of the BFS algorithm is that it can find a shortest path where the environment is known. *A*^⋆^ can also implement to find the path but it requires for a more general setting of weighted graphs. Thus, this paper prefers to use BFS as an efficient search algorithm to find the shortest path for rolling Platonic solids on 2D plane.

Based on tree traversal, BFS search has *O*(*m*^(*n*+1)^) for time complexity and *O*(*m*^*n*^) for the space complexity, which is based on figuring out size of a search tree and the number of nodes in a tree, where *m* is the maximum number of nodes in each search level and *n* is the number of layers. Depending on the type of the Platonic solids, *O*(*m*^*n*^) could be between *O*(2^*n*^) and *O*(4^*n*^) nodes in each layer. The first node *I*, which represents the initial pose, is the root of the tree, from which *m* nodes in the next layer are generated corresponding to *m* different directions of edge-rolling of each Platonic solid (*m* = 3 for tetrahedron, octahedron and icosahedron, *m* = 4 for cube, and *m* = 5 for dodecahedron) (Fig 5). From the newly generated nodes, each Platonic solid can only roll with (*m* − 1) directions to avoid going back to the previous pose in the next layer.

Nodes in the same layer representing the same pose are merged so that the algorithm only generates distinct paths. The checking condition of nodes’ orientation between the current node and the previous nodes which have the same position is added in each search iteration. This step can reduce the latter time and space searching in the main algorithm. The first path from the initial pose *I* to reach the desired pose *D* is a shortest path because of the BFS algorithm. For the stopping criteria, it is applied for only tetrahedron case when the final tetrahedron’s configuration reaches the target position but different orientation. The rest Platonic solids always reaches the target configuration. It will be explained detail next section.

### Simulation environment

**Fig 6:**
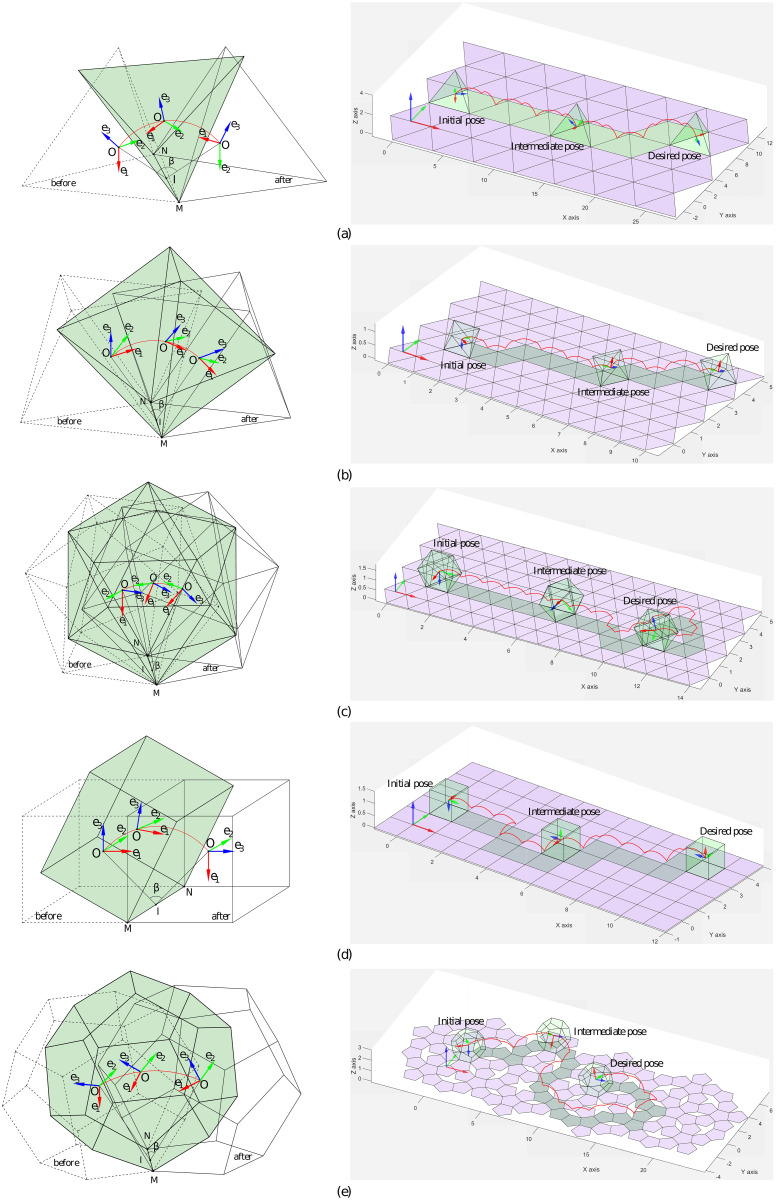
Platonic solids rolling through their edge MN with different rotation angles shown in Table 2. A body frame (***O*** − ***e***_1_ ***e***_2_ ***e***_3_) is fixed at the center of each solid (left). After edge-rolling, each Platonic solid reaches a new pose, where the red curve represents the center’s trajectory of rolling. Path-planning results for the Platonic solids on a plane shown in the right side. (a) Tetrahedron. (b) Octahedron. (c) Icosahedron. (d) Cube. (e) Dodecahedron.

We implemented the algorithm in MATLAB^®^ on a PC with a 3.6 GHz Intel Core i7 processor. A space frame was fixed at the origin and a body frame was fixed on each Platonic solid; the plane was then discretized depending on the Platonic solid (Fig 6).

## Result

This section introduces the results for the path planning of the Platonic solids on their respective grids. Fig 6(left) shows how edge-rolling changes the poses of the Platonic solids. Fig 6(right) shows the respective paths for the Platonic solids from their initial pose to their desired pose, where an additional Platonic solid shows an intermediate pose in-between.

### Tetrahedron

**Fig 7:**
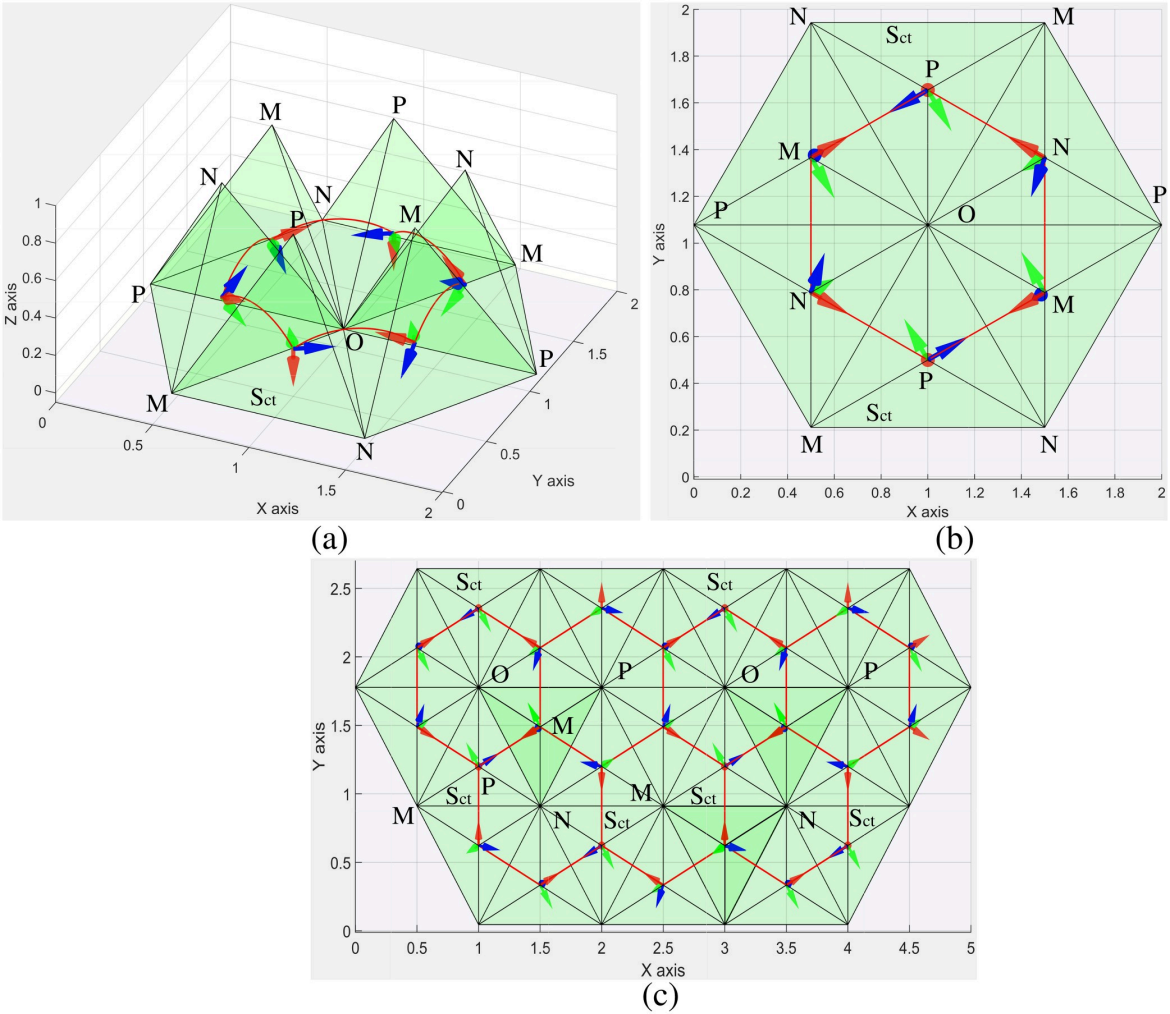
Symmetric properties of a tetrahedron. (a) A 3D view of edge-rolling 6 times around the vertex *O* where the red curve indicates the closed-path of rolling motion. (b) A top view of (a). The surface *S*_*ct*_ of the tetrahedron is in contact with the plane in different cell after sequential rolling through the edges of *NO*, *PO*, and *MO* to reach the same pose. (c) The tetrahedron reaches only one orientation for each cell through edge-rolling.

A tetrahedron is constructed from 4 incident equilateral triangles, giving 4 vertices (Table 2) and an edge-rolling angle of 2 arctan $\left(\sqrt{2}\right)$ (Table 2) on the triangular grid. The symmetry of the tetrahedron limits its reachable poses, which can be seen as follows (Fig 7). We assume the surface *S*_*ct*_ of the tetrahedron as an initial configuration (bottom of Fig 7(a) and 7(b)) is in contact with the plane where the red arrow points down to the surface contact. Because the tetrahedron has 3 incident faces at any vertex, edge-rolling along the edges *NO*, *PO*, and *MO* in sequence makes the face *S*_*ct*_ to be in contact with the plane again. Then, repeating this sequence of edge-rolling brings the tetrahedron back to the initial pose (more details in Fig 7(a) and 7(b)). As a result, the tetrahedron reaches the same pose after 6 times of edge-rolling around one vertex (Fig 7(b) and 7(c)) because the triangular grid which has 6 equilateral triangle shapes at any vertex. We conclude that only one pose can be reached for each cell starting from an initial pose due to the high symmetry brought by the tetrahedron. The shortest path for the tetrahedron is shown in Fig 6(a) (right).

### Octahedron, icosahedron, cube, and dodecahedron

On the other hand, the other 4 Platonic solids can reach an arbitrary desired pose from an initial one because the increasing number of faces impose decreasing constraints. In these cases, each position is always reached by different orientation corresponding to various paths through due to the their symmetrical properties.

An octahedron is constructed from 8 incident equilateral triangles, giving 6 vertices (Table 1) and an edge-rolling angle of arccos(1/3) (Table 2). The shortest path for the octahedron is shown in Fig 6(b) (right). An icosahedron is constructed from 20 incident equilateral triangles, giving 12 vertices (Table 1) and an edge-rolling angle of $\arccos(\sqrt{5}/3)$ (Table 2). The shortest path for the icosahedron is shown in Fig 6(c) (right). A cube is constructed from 6 incident squares, giving 8 vertices (Table 1) and an edge-rolling angle of *π*/2 (Table 2) on the square grid. The shortest path for the cube is shown in Fig 6(d) (right). Finally, a dodecahedron is constructed from 12 incident regular pentagons, giving 20 vertices (Table 1) and an edge-rolling angle of $\arccos(\sqrt{5}/5)$ (Table 2). The shortest path for the dodecahedron is shown in Fig 6(e) (right).

## Conclusion

We propose a path-planning algorithm for the Platonic solids from an initial pose to a desired pose on a plane by edge-rolling. It is straightforward to tile a plane with equilateral triangles for the tetrahedron, octahedron, and icosahedron and with squares for the cube, but there are a variety of regular pentagon tiling patterns, which all leave symmetric gaps in the plane. We chose Penrose tiling because the rhombi gaps exhibit five-fold symmetry, which facilitates the proposed algorithm. While the cube, octahedron, icosahedron, and dodecahedron can reach an arbitrary desired pose from an initial one, the tetrahedron can only reach one orientation for a cell due to the high symmetry brought by the tetrahedron and triangular grid. In this study, we successfully solved the path-planning problem of the Platonic solids by edge-rolling without obstacles on prescribed grids. We are currently investigating the possible extension of the optimal searching algorithms to more general convex solids and the optimal searching algorithm.

## Supporting information

S1 Video Tetrahedron rolling. A video of the rolling of tetrahedron following the path from an initial pose to the desired pose in Fig 6(a-right) was generated from the proposed algorithm.
(MP4)

S2 Video Octahedron rolling. A video of the rolling of octahedron following the path from an initial pose to the desired pose in Fig 6(b-right) was generated from the proposed algorithm.
(MP4)

S3 Video Icosahedron rolling. A video of the rolling of icosahedron following the path from an initial pose to the desired pose in Fig 6(c-right) was generated from the proposed algorithm.
(MP4)

S4 Video Cube rolling. A video of the rolling of cube following the path from an initial pose to the desired pose in Fig 6(d-right) was generated from the proposed algorithm.
(MP4)

S5 Video Dodecahedron rolling. A video of the rolling of dodecahedron following the path from an initial pose to the desired pose in Fig 6(e-right) was generated from the proposed algorithm.
(MP4)

## References

[1] Kline M. Mathematics in Western Culture. London: Oxford University Press; 1964.

[2] Archer-Hind R. D. The Timaeus of Plato. London: McMillan and Co.; 1888.

[3] Kepler J. Mysterium Cosmographicum. New York: Abaris Books; 1981.

[4] Damasceno P. F., Engel M., and Glotzer S. C. Predictive self-assembly of polyhedra into complex structures. Science. 2012; 337(6093):453–457. 10.1126/science.1220869 22837525

[5] Geng Y., van Anders G., Dodd P. M., Dshemuchadse J., and Glotzer S. C. Engineering entropy for the inverse design of colloidal crystals from hard shapes. Science. 2019; 5(7). 10.1126/sciadv.aaw0514 31281885 PMC6611692

[6] Cipra B. How to play platonic billiards. Science. 1997; 275(5303):1070. 10.1126/science.275.5303.1070

[7] LaValle S. M. Planning Algorithms. New York, NY, USA: Cambridge University Press. 2006.

[8] Thomas J., Blair A., and Barnes N. Towards an efficient optimal trajectory planner for multiple mobile robots. In Intelligent Robots and Systems, 2003.(IROS 2003). Proceedings. 2003 IEEE/RSJ International Conference on. 2003; 3:2291–2296.

[9] Lamiraux F. and Lammond J.-P. Smooth motion planning for car-like vehicles. IEEE Transactions on Robotics and Automation. 2001; 17(4):498–501. 10.1109/70.954762

[10] Cheng P., Shen Z., and LaValle S. M. RRT-based trajectory design for autonomous automobiles and spacecraft. Archives of control sciences. 2001; 11(3/4):167–194.

[11] Conner D. C., Rizzi A. A., and Choset H. Composition of local potential functions for global robot control and navigation. In Intelligent Robots and Systems, 2003.(IROS 2003). Proceedings. 2003 IEEE/RSJ International Conference on. 2003; 4:3546–3551.

[12] Raphael P. H., Nilsson N., and Bertram. A formal basis for the heuristic determination of minimum cost paths. IEEE Transactions on Systems Science and Cybernetics. 1968; 4:100–107. 10.1109/TSSC.1968.300136

[13] Nilsson, Nils J. Principles of Artificial Intelligence. Springer-Verlag Berlin Heidelberg. 1982; XVI, 476.

[14] Rankin A. L. and Crane III C. D. A multi-purpose off-line path planner based on an A* search algorithm. Proceedings of the ASME Design Engineering Technical Conferences. 1996; 1–10, Citeseer.

[15] Marigo A., Ceccarelli M., Piccinocchi S., and Bicchi A. Planning motions of polyhedral parts by rolling Algorithmica. 2000; 10:560–576.

[16] Erdmann M. A., Mason M. T., and Vanecek G. Mechanical parts orienting: The case of a polyhedron on a table. Algorithmica. 2005; 10:226–247.

[17] Itai A., Papadimitriou C. H., and Szwarcfiter J. L. Hamilton paths in grid graphs. SIAM Journal on Computing. 1982; 11(4):676–686. 10.1137/0211056

[18] Cromwell P. R. Polyhedra. Cambridge University Press; 1999.

[19] Dürer A. Underweysung der Messung, mit dem Zirckel und Richtscheyt, in Linien, Ebenen unnd gantzen corporen. Nüremberg: Hieronymus Andreae; 1525.

[20] Leopold C. Geometric and Aesthetic Concepts Based on Pentagonal Structures. Sriraman B., Ed. Springer International Publishing. 2019.

[21] Penrose R. Pentaplexity a class of non-periodic tilings of the plane. The Mathematical Intelligencer. 1979; 2:32–37. 10.1007/BF03024384

[22] Grünbaum B. and Shephard C. G. Tilings and Patterns. Freeman, NewYork; 1986.

[23] Dai J. S. Euler–rodrigues formula variations, quaternion conjugation and intrinsic connections. Mechanism and Machine Theory. 2015; 92:144–152. 10.1016/j.mechmachtheory.2015.03.004

[24] Cormen T. H., Leiserson C. E., Rivest R. L., and Stein C. Introduction to Algorithms Third Edition. The MIT Press; 2015.

[25] Hart P. E., Nilsson N. J., and Raphael B. A formal basis for the heuristic determination of minimum cost paths. IEEE Transactions on Systems Science and Cybernetics. 1968; 4(2). 10.1109/TSSC.1968.300136

